# Allograft as an Adjunct for Free Omental Flaps for Lower Extremity Coverage of a Mangled Lower Extremity

**DOI:** 10.7759/cureus.46146

**Published:** 2023-09-28

**Authors:** Madyson I Brown, Laura I Galarza, Nahstajia Pinnock, Jared M Davis

**Affiliations:** 1 Plastic Surgery, University of Mississippi School of Medicine, Jackson, USA; 2 Plastic Surgery, University of Mississippi Medical Center, Jackson, USA; 3 Plastic Surgery, Johns Hopkins University, Baltimore, USA

**Keywords:** trauma, microsurgery, omental free flap, allograft, lower extremity salvage

## Abstract

The utility of allograft for temporary coverage of soft tissue defects is well-established, most notably in the burn literature. Its utility as an adjunct to free tissue transfer for soft tissue defects has been described, but literature on the effectiveness of this hybrid approach for lower extremity salvage is limited.

We present a series of two patients who underwent lower extremity salvage using an omental free flap and allograft followed by staged split-thickness skin grafting at our institution. Patient characteristics analyzed included age, smoking status, comorbidities, mechanism of injury, wound class, and wound surface area. Endpoints included partial or complete flap loss, number of days from allograft to autograft, postoperative infection, unplanned reoperation, and successful, functional extremity salvage.

Both patients were male, ages 50 and 35, with a BMI of 31 and 19.2 kg/m^2^, respectively. Both were active smokers and had contaminated Gustilo IIIB wounds with areas of over 300 cm^2^. Both flaps had partial necrosis, averaging 6cm^2^, that was debrided at the planned second stage. Neither had an unplanned return to surgery, and both patients returned to ambulation.

Allograft skin as a practical and effective adjunct to omental free flap for post-traumatic lower extremity reconstruction. It can facilitate the resolution of edema and prevent flap desiccation, allowing time demarcation of partial flap necrosis and confirmation of flap viability prior to definitive skin autograft. This is particularly useful for large surface area contaminated highly irregular traumatic lower extremity wounds.

## Introduction

The omental flap was first described for free tissue transfer in 1972 [1]. Since many other flaps have been considered primary options for coverage, including muscle flaps and fasciocutaneous flaps, however, the omental flap may warrant more consideration due to its favorable anatomy, minimally invasive methods available for harvest, and other adjunctive techniques to mitigate potential shortcomings [2]. 

The omentum is a double layer of visceral peritoneum folded on itself that drapes over the contents of the abdominal cavity, and it has a long vascular pedicle with dual blood supply from the right and left gastroepiploic arteries, which branch from the gastroduodenal and splenic arteries, respectively [2,3]. The pedicle size is favorable for anastomosis to the lower leg recipient vessels end-to-end or end-to-side with an operating microscope or loupe magnification [1]. Its vasculature includes a network of arcades that allows the omentum to be partially divided and to provide coverage of large defects as a pedicled or free flap. Free tissue transfer has benefits over pedicled transfer, as it does not require a fascial or diaphragmatic defect for transposition, and it is applicable to a wider range of anatomic defects [4]. Further, the increased utilization of laparoscopic harvest has further decreased donor site morbidity, as laparotomy is not necessary. With both laparoscopic and open harvest, the flap lends well to a multidisciplinary or two-team approach, where one team harvests the flap, and another prepares the recipient site and does the microsurgery and flap inset [1,3,5,6]. The omentum has an abundance of lymphoid tissue and is highly vascular, which is beneficial for rapid revascularization, obliteration of large, irregular defects, and resolution of lymphedema [5]. It routinely exceeds an area of 300 cm^2^ in low body mass index adults and may be several times larger [4]. Further, it has been demonstrated to be favorable for re-elevation and to provide durable coverage over several years [6].

While the omental flap has several advantages, it also has qualities that make its successful use for free tissue transfer challenging. It is prone to desiccation when outside of the abdominal cavity and has a risk of partial flap necrosis, which has previously been reported to occur with a frequency of 7% in one large case series [7]. While it provides bulk and broad coverage, it requires skin grafting and can be prone to dependent edema, especially when used for lower extremity reconstruction. Still, the benefits represent significant advantages that may outweigh these in appropriately selected patients. Further, there are effective strategies available to mitigate potential pitfalls.

Many methods of coverage of free flaps devoid of skin have been described. Options include skin substitutes, non-adherent dressings, application of allograft skin, or immediate skin graft coverage. We suggest that allograft is an excellent option due to the following: cost-effectiveness compared to many skin substitutes, ability to prevent flap desiccation and insensible fluid losses, ability to maintain an environment conducive to wound healing, and decreased unplanned return to surgery for partial flap necrosis by planned staged approach [7]. For patients with burns, polytrauma, or multiple soft tissue wounds and limited donor sites, it also has the added benefit of ensuring that potential skin graft and flap donor sites are maximized. While allograft has been most commonly described for temporary coverage of burns, we believe it has a role in traumatic defects, including temporary coverage of non-skin-bearing tissue flaps such as the omental flap [8].

This report describes our experience utilizing omental free tissue transfer and allograft followed by split-thickness autograft for two devastating, large surface area lower extremity traumatic defects.

## Case presentation

Technique

This case series was exempt from IRB approval. Both patients presented with class III contaminated wounds and Gustilo IIIB lower leg orthopedic trauma. They underwent debridement, treatment of orthopedic injuries, free omental flap with immediate allograft coverage, and staged split-thickness autograft skin graft. In both cases, the greater omentum was harvested laparoscopically on the right gastroepiploic pedicle by a separate team, while the reconstructive team exposed the anterior tibial vessels. The omentum was extracted atraumatically through a 10 mm periumbilical port site and laid over the wound, with redundant area overlapped, to provide thicker coverage and better obliteration of dead space. The operating microscope was used for hand-sewn arterial and single-coupled venous anastomoses for each case. The flap was covered with cryopreserved allograft (MTF) and dressed with a bulky bolster dressing fashioned with bacitracin, vaseline gauze, and Dacron with a small window left in the dressing for visual inspection. A venous implantable Doppler and hand-held pencil Doppler were used for monitoring. The bolster was left in place until definitive skin grafting. At the planned second stage, the allograft was easily separated from the omentum with minimal bleeding, and the devitalized omentum was debrided prior to autograft application. 

**Table 1:** Patient and injury characteristics, recipient vessels, presence of partial necrosis, and follow-up time

Age	Location	Associated fracture?	Area (cm^2^)	Recipient vessels	Partial necrosis?	Follow-up time (months)
50	Right lower leg, distal third	Y	500	Anterior tibial	Y	6
35	Left lower leg distal third and dorsal foot	Y	300	Anterior tibial	Y	18

Case one

**Figure 1:**
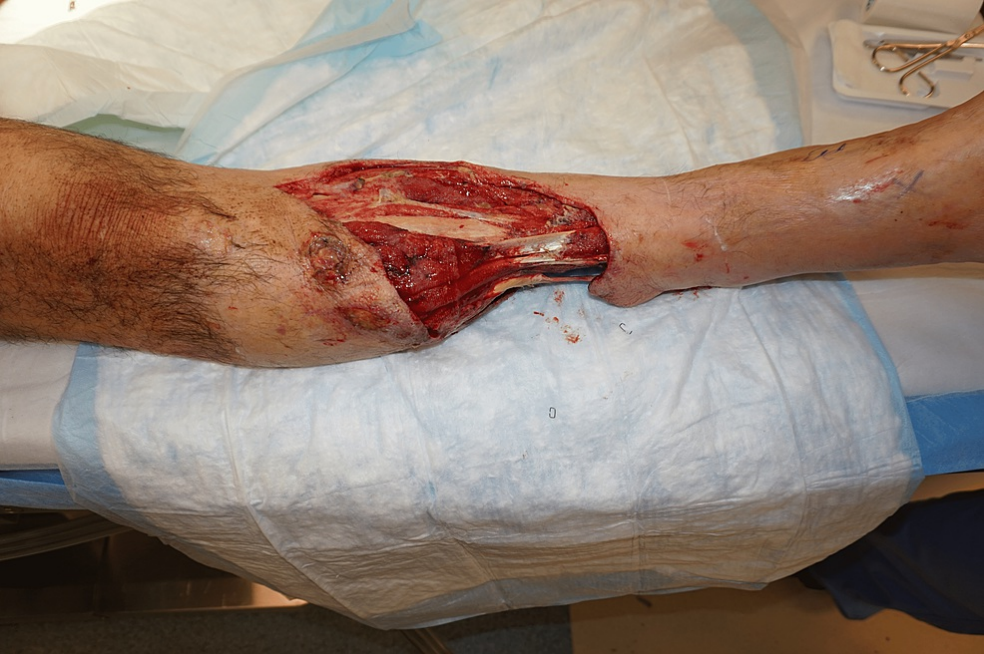
Right lower extremity near-circumferential degloving injury, anterior view

**Figure 2:**
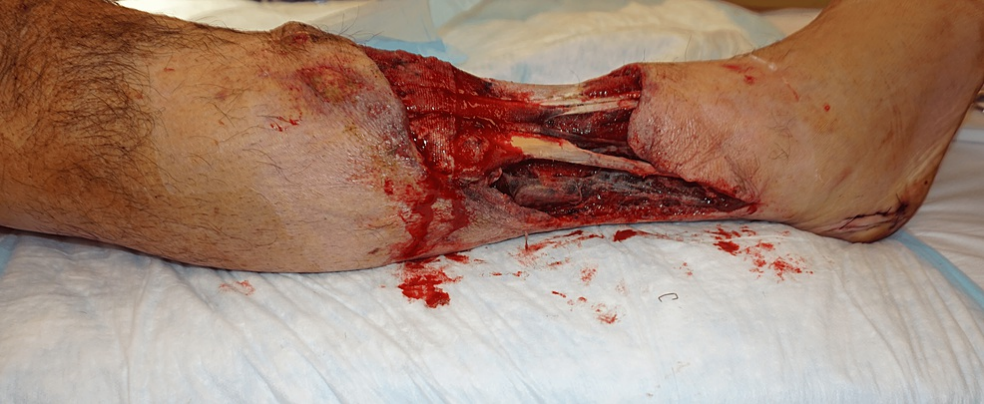
Right lower extremity near-circumferential degloving injury, lateral view

A 50-year-old man with a history of previous substance abuse, active tobacco abuse, and a BMI of 31 presented with bilateral lower extremity crush-avulsion injuries following an industrial farming accident and prolonged extrication. He suffered mangled bilateral lower extremities with multilevel bone, vascular, and nerve injuries on the left distal thigh and lower leg and presented with a tourniquet on the left proximal thigh. The left lower extremity underwent revision above knee amputation. The right lower extremity had degloving of the distal half of the lower leg (Figures 1, 2), segmental loss of the fibula, and single vessel runoff to the foot. Both wounds were grossly contaminated. 

**Figure 3:**
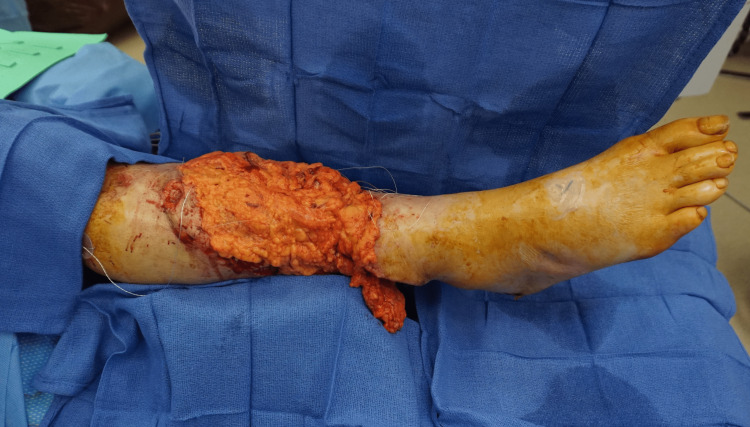
Free omental flap after inset and anastomosis and prior to allograft application

**Figure 4:**
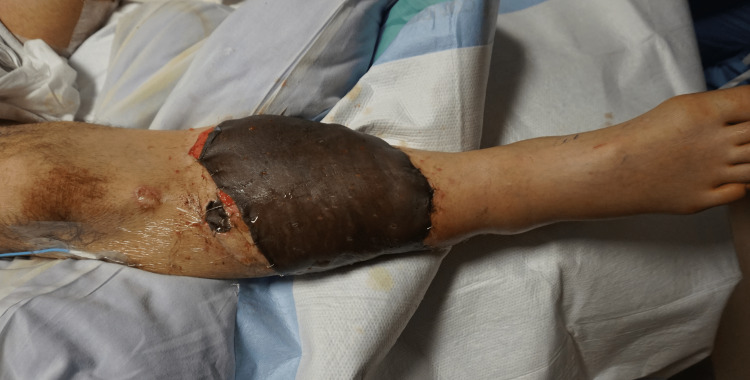
Free omental flap, prior to debridement of allograft and coverage with autograft

**Figure 5:**
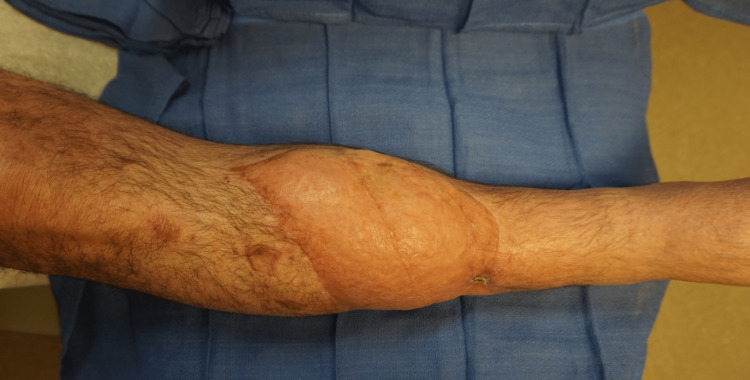
Free omental flap at the three-month postoperative visit

The right lower leg underwent salvage, free omental flap (Figure 3), and allograft coverage (Figure 4) of the 500 cm2 wound. His postoperative care was complicated by post-traumatic stress disorder (PTSD) upon emergence from anesthesia. This resulted in a delay of several days for psychiatric optimization, rather than proceeding with the second stage two to three days after the index operation as planned. On postoperative day nine, the patient returned for planned, staged allograft debridement and skin autograft. The allograft was excised, and a distal 4 cm^2^ segment of the omental flap was noted to be necrotic. The non-viable end of the flap not overlying any critical structures and was debrided, followed by placement of a fenestrated sheet split-thickness skin autograft. The postoperative course was uneventful, and the patient was fitted with a left above-knee prosthesis after healing of the above knee amputation stump. He regained ambulatory function, returned to work, and did not require any further surgery (Figure 5). 

Case two

**Figure 6:**
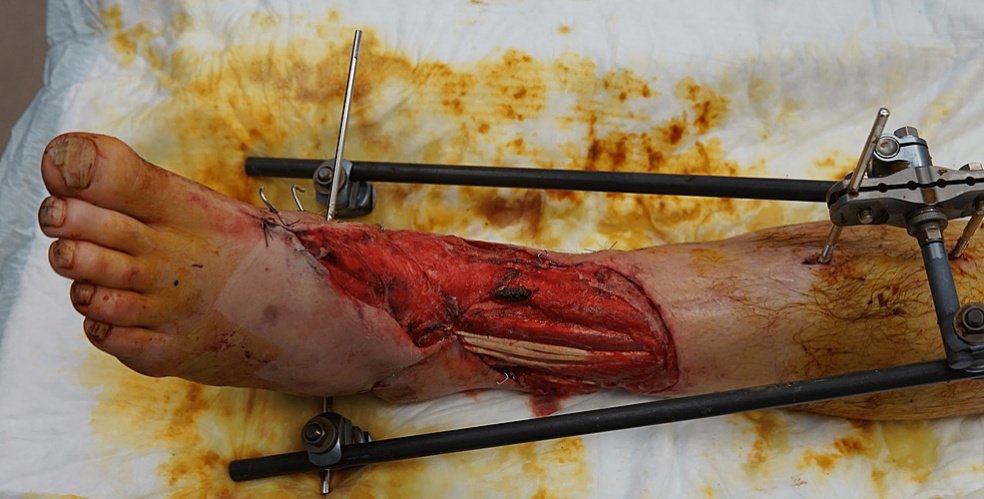
Left lower extremity open tibial fracture with degloving injury, status post external fixation and bone cement spacer placement

**Figure 7:**
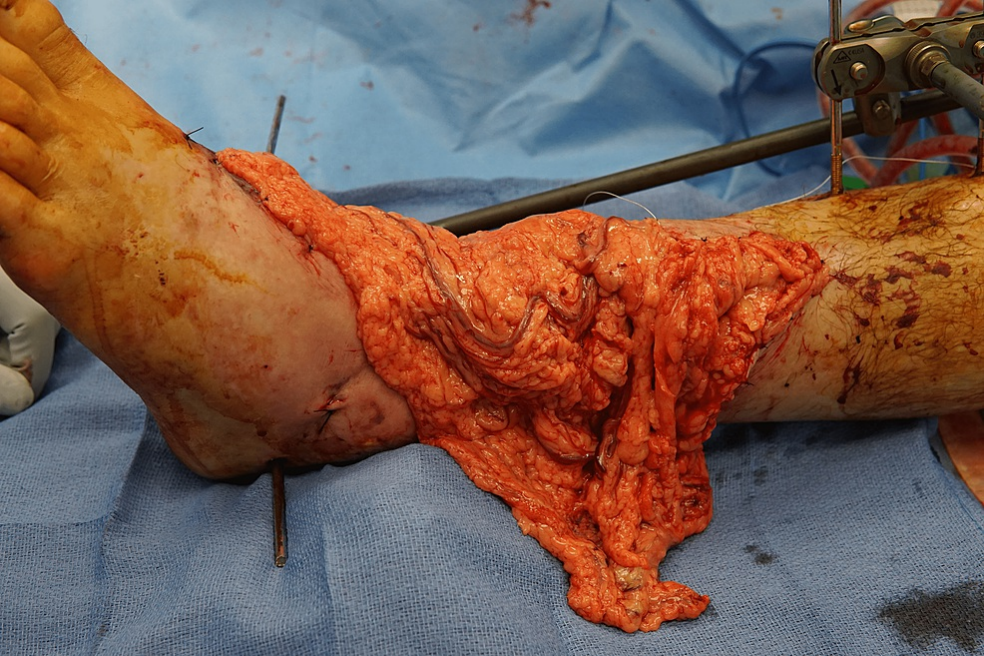
Free omental flap. Note that the external fixator was partially deconstructed to facilitate microsurgical anastomosis

A 35-year-old man with a history of tobacco abuse presented as a pedestrian struck by an automobile with contaminated degloving injury of the left anterior ankle and dorsal foot with two vessel runoff to the foot. He underwent external fixation of the tibial fracture with cement spacer placement (Figure 6) and free omental flap (Figure 7) with temporary allograft coverage. He returned to the operating room on postoperative day three for a split-thickness fenestrated sheet autograft. After allograft debridement, there were two small areas totaling 8 cm^2^ of devitalized omentum that were debrided prior to fenestrated split thickness sheet grafting. 

**Figure 8:**
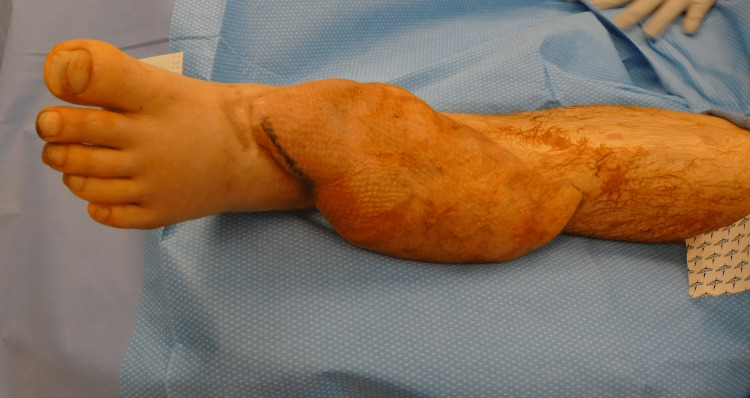
Omental flap prior to debulking one year postoperatively

His postoperative course was complicated by a suspected hardware infection that was treated with intravenous antibiotics and removal of the external fixator. He subsequently underwent flap elevation, bone cement removal, and bone grafting via the Masquelet technique with simultaneous flap debulking. He regained ambulatory function, albeit with flap edema with prolonged standing. He underwent further debulking at one year after the index operation for edema with prolonged standing (figure 8).

## Discussion

Lower extremity salvages present reconstructive surgeons with unique challenges, and thus, careful decision-making is essential. It is important to consider the defect, patient comorbidities, smoking status, overall trauma burden, donor site morbidity, and need for future surgery when selecting reconstructive modalities and donor sites. The severity of the bony and soft tissue injuries, wound surface area, and the need for dead space obliteration dictate the most appropriate options for free tissue transfer. The omental flap is useful for coverage of a variety of defects, including for thoracic, head and neck, and extremity applications [1-4,7]. The omentum possesses unique immunologic and angiogenic properties that make it favorable for the prevention of infection and lymphedema, two sources of immediate and delayed morbidity in mangled lower extremities, especially in contaminated, degloved lower extremities. Mazzafero et al. discussed the utility of omental-free flaps for the management of lymphedema, both with and without lymphovenous anastomosis [2]. In essence, it can serve as a functional lymph node transfer. Their review included six studies and 37 patients, including seven patients who had traumatic etiology of wounds. Of these, six of the seven flaps survived. Other authors have previously reported staged split-thickness skin grafting; however, to our knowledge, the use of allograft skin for temporary coverage of a free omental flap for coverage of traumatic lower extremity defects has not been described [9].

The main drawbacks of the omental flap include the need for laparotomy or laparoscopy for harvest and its tendency for distal flap necrosis and desiccation without early, appropriate coverage. The morbidity associated with flap harvest has been reduced as laparoscopy has become more widely used for harvest [4,5]. We suggest that the two other drawbacks are mitigated by using allograft skin for temporary coverage at the time of flap elevation and inset. Allograft decreases insensible fluid losses, prevents flap desiccation, and prepares wounds for autologous skin graft [10]. It also provides physiologic coverage, similar to autograft, without donor site pain and additional surgical time, and protects the flap while potential areas of partial necrosis demarcate. Planned return for staged autograft provides an opportunity to address distal flap necrosis with excision and readvancement of the flap, if necessary. While the distal edges of the flap can be trimmed at the initial inset, imbricating the omentum on itself allows for more durable, thick coverage, especially in lower BMI patients whomay have a thinner omentum. In our experience, the allograft is able to be easily separated from the omental flap without significant bleeding or trauma. While a period of two to three days is more than adequate to allow for demarcation, our experience demonstrates that allograft separation is feasible with minimal flap trauma up to nine days after application.

## Conclusions

In summary, a hybrid technique utilizing the omental flap and allograft, followed by split-thickness autograft, is a powerful option for coverage of contaminated, large, degloving injuries and orthopedic trauma of the lower leg. While a review of the various skin substitutes is beyond the scope of this discussion, it is important to be aware of other options that are available for temporary coverage. While pricing varies among institutions, allograft is a cost-effective method of coverage. Staging of the omental flap and definitive skin graft coverage is prudent to avoid unplanned reoperation or other complications resulting from partial necrosis. 
